# Patient satisfaction, joint stability and return to sports following simple elbow dislocations: surgical versus non-surgical treatment

**DOI:** 10.1007/s00402-022-04383-8

**Published:** 2022-02-26

**Authors:** Stephanie Geyer, Lucca Lacheta, Jesse Seilern und Aspang, Lukas Willinger, Patricia M. Lutz, Sebastian Lappen, Andreas B. Imhoff, Sebastian Siebenlist

**Affiliations:** 1 Department of Orthopaedic Sports Medicine, Technical University of Munich, Ismaninger Straße 22, 81675 Munich, Germany; 2 Department of Shoulder and Elbow Surgery, Center for Musculoskeletal Surgery, Charité-Universitätsmedizin Berlin, Corporate Member of Freie Universität Berlin and Humboldt-Universität zu Berlin, Campus Virchow, Augustenburger Platz 1, 13353 Berlin, Germany; 3 Department of Orthopaedics, Emory University, Atlanta, GA USA; 4 Department of Trauma Surgery, Technical University of Munich, Ismaninger Straße 22, 81675 Munich, Germany

**Keywords:** Elbow, Simple elbow dislocation, Instability, Ultrasound, Return to sports

## Abstract

**Purpose:**

While conservative management is commonly promoted for simple elbow dislocations, the importance of primary surgical treatment in these injuries is still undetermined. The objective of this study was to report patient-reported outcome measures (PROMs), return to sports (RTS) and joint stability using ultrasound in patients following conservative or surgical treatment after simple elbow dislocation.

**Methods:**

Patients with a minimum follow-up of 24 months after conservative (CT) or surgical treatment (ST) following simple elbow dislocation were included in this retrospective study. To evaluate patients’ postoperative outcome and satisfaction, the Elbow Self-Assessment Score (ESAS) was used, and validated scores such as the Mayo elbow performance score (MEPS), the Quick Disability of Arm and Shoulder Score (Quick-DASH) and RTS were assessed. For objective assessment of residual joint instability, a standardized clinical examination as well as a dynamic ultrasound evaluation of the affected and the contralateral elbow was performed.

**Results:**

Forty-four patients (26 women, 18 men) with an average age of 41.5 ± 15.3 years were available for follow-up survey (65.5 ± 30.4 months; range 26–123). 21 patients were treated conservatively and twenty-three patients received surgical treatment. CT and ST resulted in similar outcome with regard to ROM, ESAS (CT: 99.4 ± 1.5; ST: 99.8 ± 0.3), MEPS (CT: 97.3 ± 6.8 points; ST: 98.7 ± 3.3) and Quick-DASH (CT: 7.8 ± 10.4; ST: 6.3 ± 7.9) (n.s.). There was no difference in elbow stability and laxity measured by ultrasound between the study groups and compared to the healthy elbow (n.s.). Two patients of the CT group (10%) complained about persistent subjective elbow instability. RTS was faster after surgical compared to conservative treatment (*p* = 0.036).

**Conclusion:**

Both, conservative and surgical treatment results in high patient satisfaction and good-to-excellent functional outcome after simple elbow dislocation. Even though ultrasound evaluation showed no significant differences in joint gapping between groups, 10% of conservatively treated patients complained about severe subjective instability. Surgically treated patients returned faster to their preoperatively performed sports. Thus, primary surgical treatment may be beneficial for high demanding patients.

**Level of evidence:**

Therapeutic study, Level III.

## Introduction

With an incidence of 6–9/100′000, the elbow is the second most frequently dislocated joint in humans after the shoulder [1, 24]. In general, two types of elbow dislocation injuries can be distinguished. The “simple” (= ligamentous) dislocation includes injury to collateral ligaments, capsule and tendon/muscle insertions with bony avulsion ≤ 2 mm, while “complex” dislocations include additional bony lesions of the ulna, radius and/or humerus [11]. Up to 75% of elbow dislocations are simple elbow dislocations [8].

A vast majority of authors consider conservative management for these soft tissue injuries the standard of care, encompassing initial immobilization and early functional physical therapy [14, 15, 19, 20, 22, 26]. Nevertheless, complaints of stiffness, pain and instability remain in 10% of conservatively treated cases [1, 16].

With the increasing number of retrospective studies reporting promising results after surgical capsulo-ligamentous repair and musculo-ligamentous refixation, primary surgical management of elbow dislocations has become a pertinent topic of discussion [4, 7, 8, 18, 21, 23, 25]. In a systematic review, Hackl et al. [6] summarized that given the paucity of randomized controlled trials on this topic, further research with focus on subjective patients’ outcome such as pain, reduced range of motion (ROM) and persistent instability is warranted to clarify the importance of surgery after ligamentous elbow dislocation.

The purpose of this study was to (1) determine patient satisfaction, patient-reported outcome measures (PROMs), return to sports rate (RTS) and functional elbow scores following conservative vs. surgical treatment of ligamentous elbow dislocation, and to (2) evaluate subjective and objective joint stability. It was hypothesized that (1) outcome measures would result in comparable clinical outcomes between both treatment groups, but that (2) CT patients would exhibit increased joint gapping during ultrasound evaluation.

## Materials and methods

### Study population

A retrospective chart review was performed to identify patients who were treated for simple elbow dislocations in an academic Level-1 trauma center between April 2010 and April 2018. Institutional review board approval was obtained for the study (65/16S) and it was conducted according to the Declaration of Helsinki. Informed written consent was obtained from each patient.

All patients with a ligamentous elbow dislocation involving soft tissue injury of ligaments, capsule and/or tendons and muscles with bony avulsion ≤ 2 mm were included with a minimum follow-up of 24 months. Patients with a complex elbow dislocation including bony avulsion > 2 mm, radial head and/or olecranon fracture, coronoid process fracture, as well as patients aged younger than 18 years were excluded. Individuals with prior elbow injuries, patients who received secondary ligament reconstruction and patients with frailty due to vascular diseases or dementia were also excluded from the present survey.

Eligible patients were contacted and invited to participate a standardized clinical and ultrasound examination in our clinic. Posttraumatic 3-Tesla MRI were screened and injury patterns were recorded for analysis.

### Treatment

**Fig. 1:**
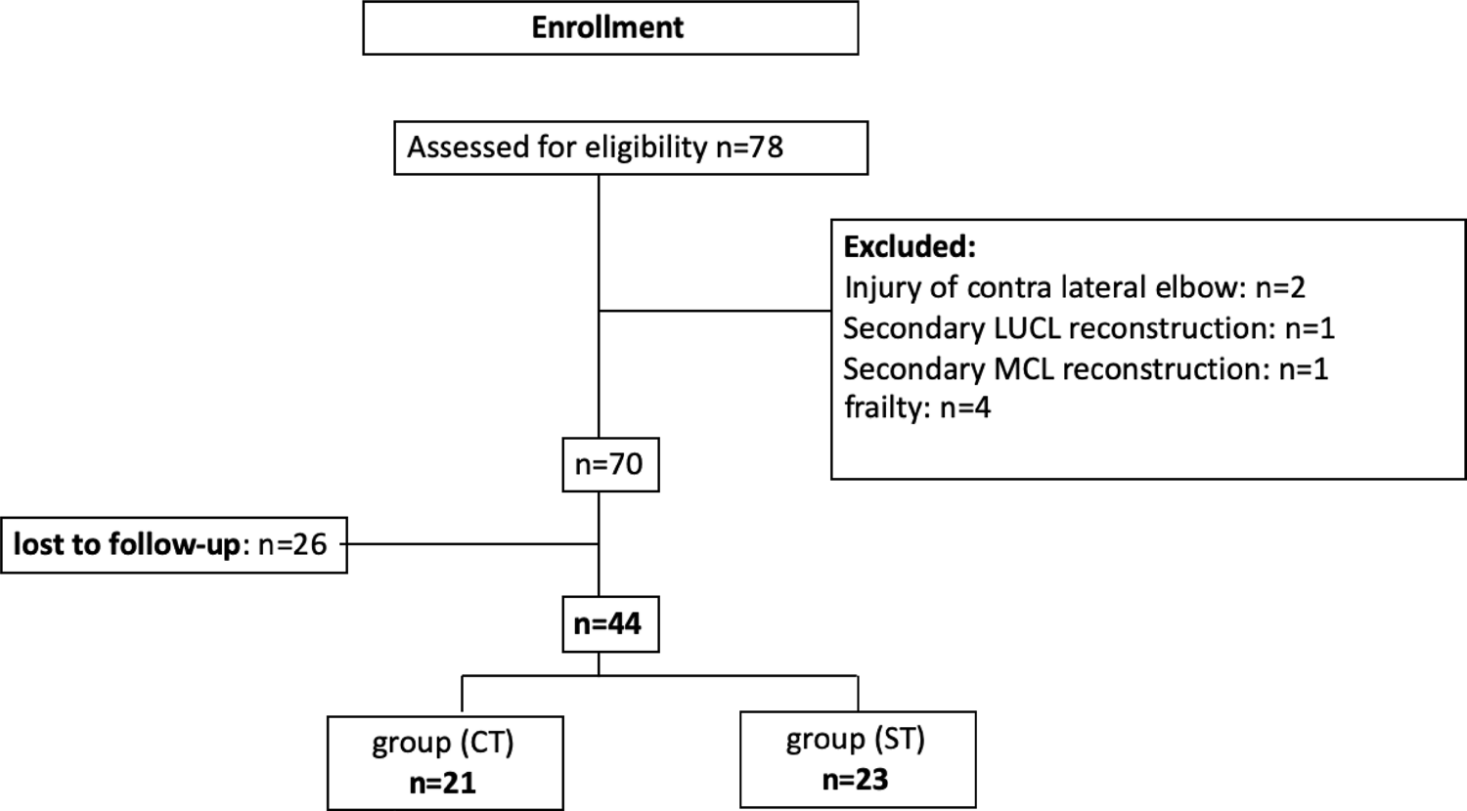
Study population. (LUCL, lateral ulnar collateral ligament; MCL, medial collateral ligament complex; CT, conservative treatment; ST, surgical treatment)

Patients were grouped according to the treatment received in either CT (= conservative treatment) or ST (= surgical treatment) (Fig. 1). CT was indicated for patients if elbow joint congruency was confirmed under fluoroscopy after closed reduction within the functional arc of motion (between extension/flexion 0°–30°–130°). Patients who initially showed evidence of subluxation or re-dislocation during fluoroscopy, joint incongruency in subsequent control radiographs (positive drop sign with ≥ 4 mm), and/or had high functional demands received capsule–ligamentous repair and tendon refixation (ST group). Depending on clinical assessment of medial, lateral and posterolateral instability and correlating soft tissue damage on MRI, the decision was made for unilateral or bilateral approach by the surgeon.

Patients of both groups received a standardized rehabilitation program. A hinged elbow brace was applied in all patients for six weeks after injury/surgery. In addition, ST patients were initially immobilized with an above elbow backslab for two days after surgery in 90° flexion. ROM was limited to extension/flexion 0°–20°–110° for four weeks postoperatively and was thereafter unrestricted. Patients underwent supervised physical therapy including overhead motion exercise for at least six weeks after trauma/surgery.

### Clinical outcome assessment

The functional results were assessed by the Mayo elbow performance score (MEPS), Quick Disabilities of Arm and Shoulder Score (Quick-DASH) and Elbow Self-Assessment Score (ESAS). The ESAS Score consists of 22 items and is a subjective assessment of elbow function, designed to provide a valid statement of the patients’ level of satisfaction [2]. A visual analog scale (range from 0 to 10; 0 = no instability; 10 = completely unstable elbow) was used to assess subjective persistent elbow instability. Besides pain and limitations in daily life activities—elbow ROM with flexion/extension as well as pronation/supination and subjective feeling of stability/instability were assessed in a standardized fashion. All patients underwent clinical assessments at final follow-up survey. ROM was measured with a goniometer and stability was tested with a varus and valgus stress test in 0° and 30°. Furthermore, the pre- and postoperative activity level, the amount and the sports performed and the time from dislocation to return to sports were assessed.

### Ultrasound-assisted stability testing

**Fig. 2:**
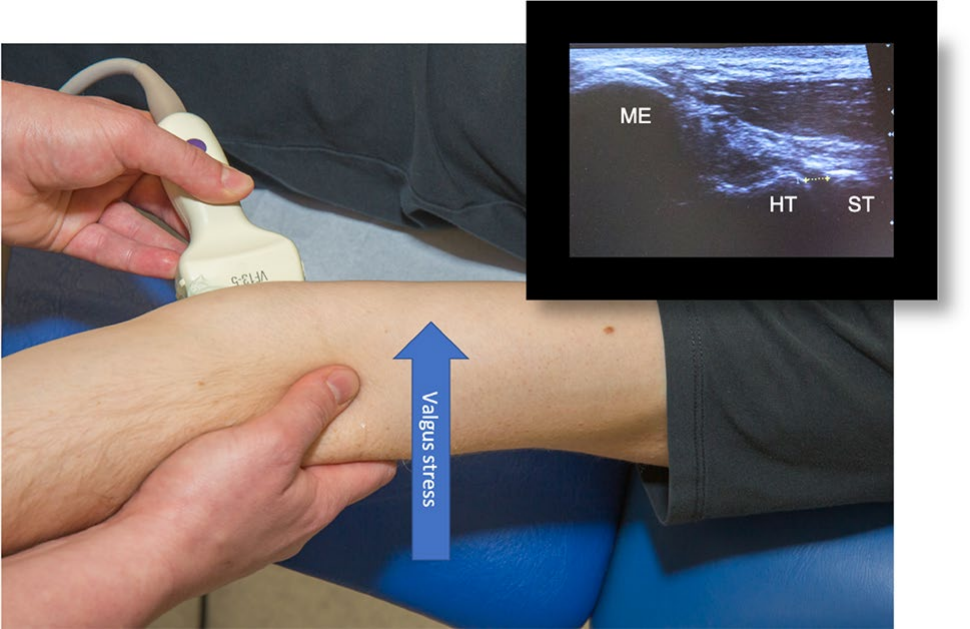
Patient in supine position, ultrasound of the medial left elbow (landmarks: medial epicondyle (ME); humeral trochlea (HT) and sublime tubercle (ST). Valgus stress testing in full extension measuring the joint space between the HT and the ST

**Fig. 3:**
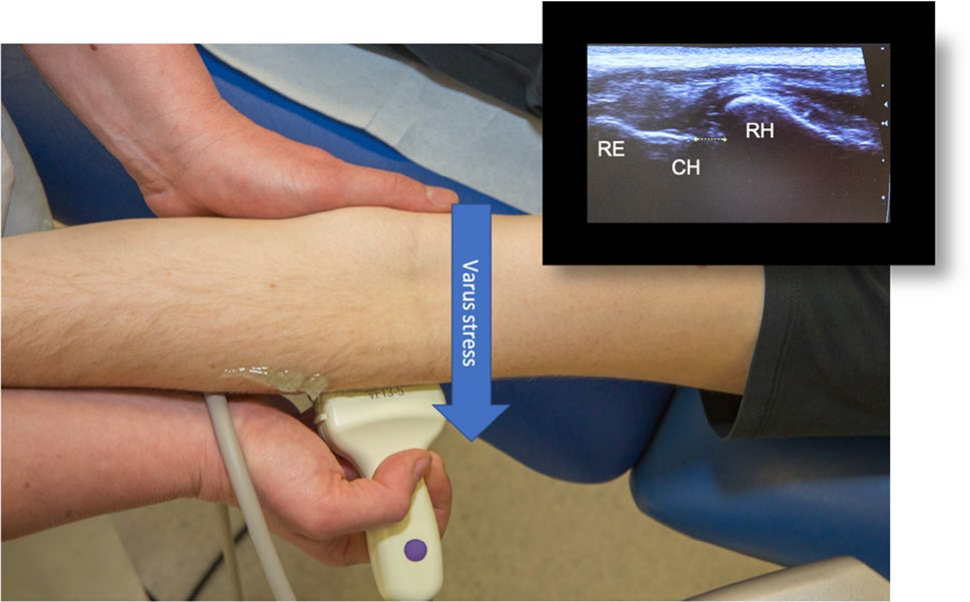
Patient in supine position, ultrasound of the lateral left elbow (landmarks: radial epicondyle (RE), capitulum humeri (CH), radial head (RH)) with focus on the joint line. Varus stress testing in full extension measuring the joint space between the CR and the RH

A standardized ultrasound examination with a 13.5 MHz linear transducer was performed on both elbow joints with the ACUSON X300 ultrasound device (ACUSON X300, Premium Edition, Siemens Inc., Mountain View, USA). All patients were examined in supine position with a fully supinated elbow joint. Measurements were recorded for both elbows in 0° and 30° of flexion. The ultrasound examinations were performed by an experienced senior orthopedic resident. First, the transducer was placed parallel to the fibers of the anterior bundle of the medial collateral ligament (MCL) to visualize the medial joint space between the humeral trochlea and sublime tubercle. The joint space was measured by ultrasound with and without manually applied valgus stress at 0° and 30° flexion (Fig. 2). The lateral joint space was quantified likewise by measuring the coronal distance between the capitulum and the radial head with and without manually applied varus stress at 0° and 30° flexion (Fig. 3). Measurements were compared to the healthy contralateral elbow. Elbow joint laxity was defined as the increase in joint space when load was applied and compared to the native elbow laxity of the uninjured elbow in each patient.

### Statistical analysis

A priori power analysis was conducted using G*Power software, requiring a sample size of 43 for a significance level of 1% (*p* < 0.01) and power of 80% [10]. All calculations were performed with SPSS Statistics (IBM Corp. Released 2019, Version 26.0. Armonk, NY, USA). Statistical means, minimum, maximum and standard deviations were calculated for continuous variables. The Fisher’s exact test was applied to compare the distribution of MRI injury pattern between conservative and surgical therapy. The Mann–Whitney-*U* Test and the Wilcoxon were used to compare CT with ST as nonparametric test for the null hypothesis according to the performed evaluation of normal distribution. The Pearson correlation was used to test the correlation between subjective instability rated on VAS and the ultrasound joint gapping as well as the assessed scores. Statistical significance was defined as *p* < 0.05.

## Results

70 patients who suffered from acute ligamentous elbow dislocation were identified and met the inclusion criteria. Of eight excluded patients (Fig. 1), two received secondary ligament reconstruction (*n* = 1 LUCL and *n* = 1 MCL) due to presenting persisting instability following conservative treatment. The final study population consisted of 44 patients (26 females/18 males) with a mean age of 41.5 ± 15.3 years. 26 patients were lost to follow-up (18 group CT/8 group ST): 18 due to unknown address, three due to remote locations, two due to personal schedule conflicts and three patients did not want to participate. The mean follow-up period was 65.5 ± 30.4 months (range 26–123). Out of the 44 patients, 21 patients were treated conservatively, and 23 patients were treated surgically. The demographics of the two groups are summarized in Table 1. No statistical difference concerning the demographic data was found between the two groups.

**Table 1:** Demographic data for CT (conservative treatment) vs. ST (surgical treatment) group

Demographics	Group CT (*n* = 21)	Group ST (*n* = 23)
Age [years, mean ± SD]	37.4 ± 16.2	42.6 ± 14.1
Sex [f/m]	13/8	13/10
BMI [kg/m^2^, mean ± SD]	23.9 ± 2.6	24.3 ± 3.4
Dominant arm affected	9	9
Follow-up, mean ± SD [months]	72.8 ± 33.6	58.7 ± 26.1
Performing sports prior to injury (%)	16 (76%)	21(91.3%)

SD, standard deviation; f, female; m, male; BMI, body mass index; kg, kilogram; m, meter

Nine of the 21 patients in group CT and all of group ST patients had undergone MRI evaluation. The soft tissue injury patterns demonstrated on MRI are summarized in Table 2. Patients with a complete lesion of the collateral ligaments (MCL, LCL/LUCL) and concomitant lesion of flexors and/or extensors were more likely to receive surgical treatment but without reaching statistical significance.

**Table 2:** Soft tissue injury patterns according to preoperative magnetic resonance imaging

Injury	Group CT (*n* = 9)	Group ST (*n* = 23)	*p*-value
MCL^a^ and flexors^c^	2	2	0.568
LCL/LUCL^b^ and extensors^d^	0	1	0.876
MCL^a^, LCL/LUCL^b^ without flexors^c^ and extensors^4^	2	1	0.532
Partial rupture of MCL^a^ and LCL/LUCL^b^ in combination with flexors^c^ and/or extensors^d^	2	3	0.527
Complete rupture of MCL^a^ and LCL/LUCL^b^ in combination with flexors^c^ and/or extensors^d^	3	16	0.061

n.s., not significant

^a^Medial collateral ligament

^b^Lateral collateral ligament/lateral ulnar collateral ligament

^c^Common flexor tendon

^d^Common extensor tendon

Surgery was performed by three surgeons after an average of 8.7 ± 3.7 days and were as follows: 16 patients underwent combined LUCL- and MCL repair, 1 patient LUCL repair and extensor refixation and six patients MCL repair and flexor refixation, respectively. In all cases capsulo-ligament repairs were performed with suture anchors (Arthrex Inc., Naples, FL, USA). In two patients additional internal bracing was performed for LUCL repair. 11 patients underwent additional neurolysis of the ulnar nerve.

### Clinical outcome

At follow-up survey, the mean ESAS reached 99.6 ± 1.1 points, the MEPS 98.2 ± 5.2 points and the Quick-DASH was 7.0 ± 9.1 points in all patients. Subgroup analysis (CT vs. ST) of the clinical scores did not show a significant difference and are summarized in Table 3. However, the VAS for instability did correlate significantly with the ESAS (*p* = 0.000), the MEPS (*p* = 0.000) and the Quick-DASH (*p* = 0.006). MEPS was rated as “excellent” in *n* = 42 (95.5%), as “good” *n* = 1 (ST group) and as “fair” *n* = 1 (CT group) according to Nestor[17].

**Table 3:** Summary of patient-reported outcomes (PROs), functional scores and arc of motion

Score	Group CT	Group ST	*p*-value
ESAS^a^	99.4 ± 1.5	99.8 ± 0.3	0.625
MEPS^b^	97.3 ± 6.8	98.7 ± 3.3	0.222
Quick-DASH^c^	7.8 ± 10.4	6.3 ± 7.9	0.766
Extension/flexion (°)	138.7 ± 8.3	135.4 ± 13.7	0.845
Pro-/supination (°)	180 ± 0	177.4 ± 7.5	0.663

(°), degree

^a^Elbow Self-Assessment Score (ESAS)

^b^Mayo elbow performance score (MEPS)

^c^Quick Disability of Arm and Shoulder Score (Quick-DASH)

ROM of the affected elbow was significantly smaller compared to the healthy side in the CT group (*p* = 0.005) and the ST group (*p* = 0.001) but not different between the treatment groups (Table 3). Eight patients showed an extension deficit ≥ 5°–15° (CT *n* = 4; ST *n* = 4), whereas ten patients showed a persisting flexion deficit ≥ 10° (CT *n* = 4; ST *n* = 6).

Four CT patients complained about persistent subjective instability sensations (VAS for instability 4.8, range 1–10); two of them (10%) about severe elbow instability. Six ST patients reported only mild subjective instability (VAS for instability 1.5, range 1–2).

### Ultrasound-assisted stability testing

All patients demonstrated significant changes in ligament length in millimeters appraised by stress testing for the affected and non-affected elbow at 0° and 30° flexion (*p* < 0.001).

**Fig. 4:**
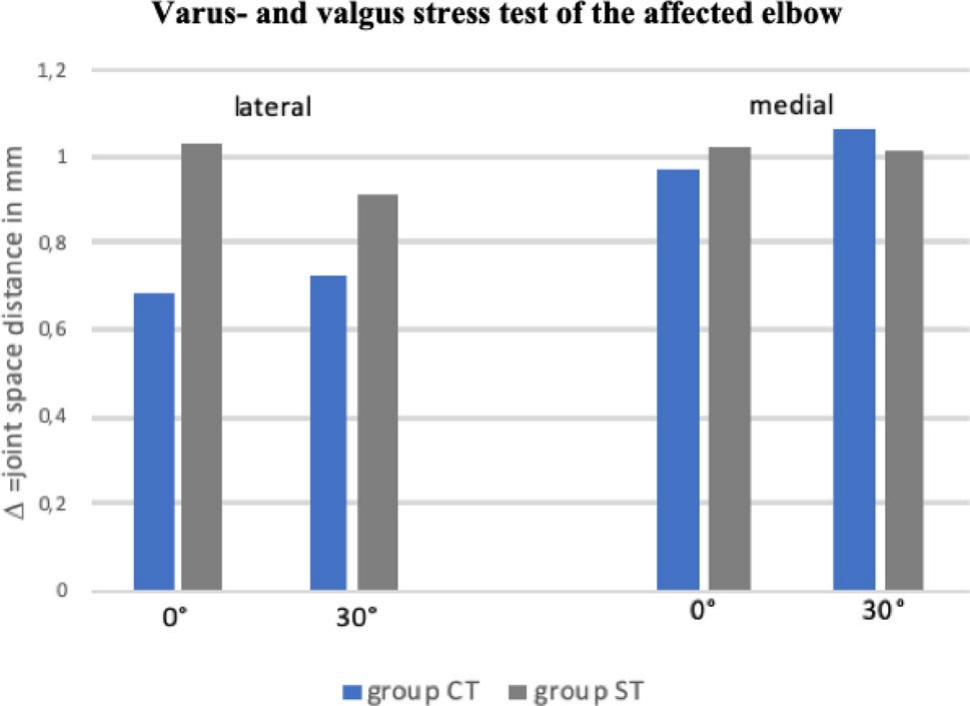
Varus- and valgus stress test of the affected elbow comparing group CT and ST. Zero represents the native joint space (in mm) without applied valgus (for medial side) or varus (for lateral side) stress

**Fig. 5:**
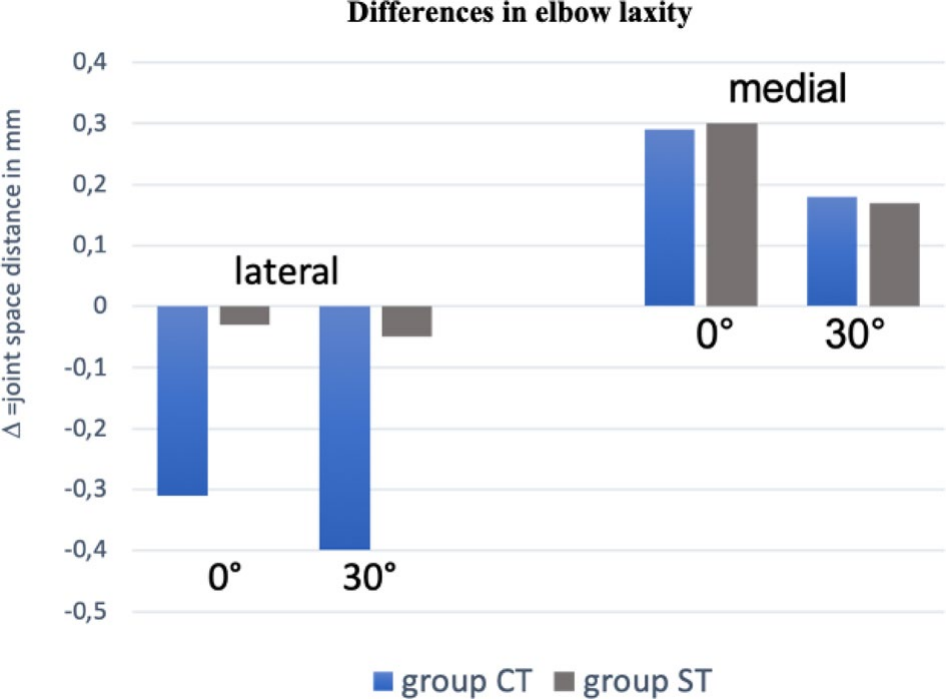
Delta of the differences in elbow laxity obtained by ultrasound stability testing between injured and healthy contralateral elbow. Zero represents the native elbow laxity of the uninjured side

Comparing joint space opening of the affected and unaffected elbow with applied varus/valgus stress did not show a significant difference at 0° and 30° flexion (n.s.). CT and ST resulted in comparable elbow stability and in similar objective joint laxity compared to the healthy elbow (both n.s., Figs. 4 and 5).

Subjective instability measured on a VAS scale (0–10) showed no significant correlation with the joint gap widening during ultrasound test (Delta stress test) (n.s.). However, out of 10 patients complaining of subjective instability (CT: *n* = 4; ST: *n* = 6), 4 patients also revealed objective instability by the delta stress test result, with *n* = 1 and *n* = 3 for CT and ST patients, respectively.

### Return to sport (RTS) after simple elbow dislocation

**Fig. 6:**
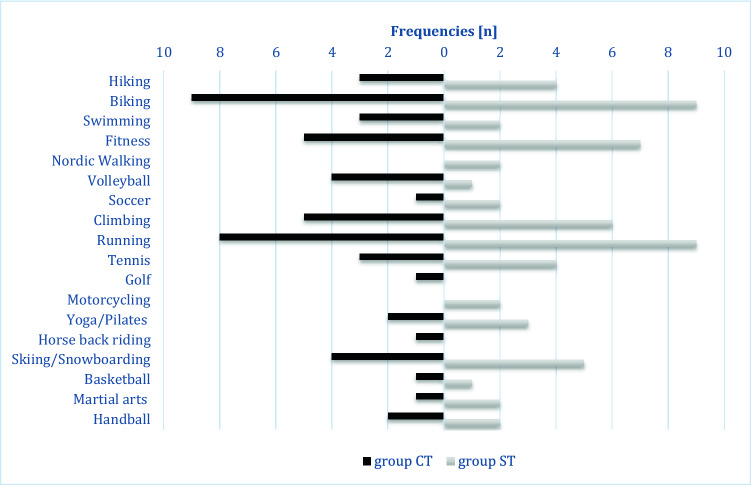
Frequencies of sportive activity of both groups before elbow dislocation [*n*], multiple answers were possible

100% of pre-injury active patients (CT: 16/21 (76.0%), ST: 21/23 (91.3%)) returned to sports regardless of the treatment received. All patients who didn’t perform sports previously remained non-sportive after treatment. Patients in ST returned to sports significantly faster (mean 3 ± 4.9 months; range, 2–24) than patients in CT (6 ± 20.4 months; range 3–84; *p* = 0.036). Frequencies of sportive activity before simple elbow dislocation of CT and ST patients are summarized in Fig. 6.

Three patients of the CT group played sports daily, nine patients twice or more frequently per week and three once a week. In the ST group four patients played daily, twelve twice or more per week, four once per week and one played sport once per month. After simple elbow dislocation one patient in group CT and one patient in group ST performed sports less often due to elbow-unrelated reasons. 25 patients (9 group CT; 16 group ST) felt unchanged activity for their sportive activities and six patients reported impairment in group CT (40%) and five in group ST (23.8%). In group CT, eight patients remained very satisfied with their level of sportive activities, six were somewhat satisfied, one somewhat unsatisfied and no patient unsatisfied and in group ST 17 remained very satisfied and four somewhat satisfied. In group CT, four patients (26.7%) reported pain while playing sports with a mean VAS of 1.6 ± 1.2. For group ST, three patients (14.3%) complained about pain with a mean VAS of 1.4 ± 1.1. In group CT, three patients continued sports but gave up tennis (2) and snowboarding (1). In group ST, three patients continued sports but one gave up motorcycling and martial arts due to pain, one gave up handball and cheerleading due to the recommendation of the surgeon and one handball due to elbow-unrelated reasons.

### Complications

The overall complication rate for group CT was 9.5%. The complication and revision rate for ST patients was 21.7% and 8.7%, respectively (n.s.). One patient of the initially conservative treated patients showed progressive joint incongruency with a re-dislocation after three days and needed secondary surgical treatment (4.8%). Transient ulnar nerve syndrome was documented in two patients (both group CT 9.5%). A prolonged range of motion deficit longer than six months was noted in three ST patients (13.0%). In those, revision surgery was not required since ROM increased to full range of motion within twelve months after trauma. Two ST patients underwent revision surgery (8.7%). One patient received an arthroscopic arthrolysis 10 months after primary surgery due to persistent ROM deficit. One patient demonstrated reduced ROM (extension/flexion 0°–70°–80°) due to heterotopic ossifications. Open arthrolysis and ulnar nerve decompression was performed five months after the initial trauma. No infections occurred in the entire study cohort.

## Discussion

The most important findings of the present study were that high patient satisfaction, excellent clinical results in 95.5%, and no significant difference for ultrasound measured joint gapping was achieved following simple elbow dislocation, regardless of the treatment (conservative vs. surgical therapy). The objective measurement of elbow laxity did not correlate with the subjective impression of persistent joint instability of the patients. However, patients with a complete lesion of the collateral ligaments (MCL, LCL/LUCL) and concomitant lesion of flexors and/or extensors were more likely to receive a surgical approach. Moreover, patients undergoing surgical musculo-ligamental reconstruction achieved a faster return to sports with a higher rate of satisfaction while performing sports after trauma. However, there was a tendency for a higher complication rate in surgically treated patients.

There is a lack of well-designed randomized controlled trials comparing conservative with surgical treatment following simple elbow dislocation [6]. Josefsson et al. [10], published a randomized controlled trail with two subgroups: conservative treatment (*n* = 15) and surgical treatment (*n* = 15) without assessing clinical scores such as the MEPS or DASH score. The authors found similar outcome for ROM and re-instability rate. This is in agreement with the results of the present study with regard to ROM but we recognized a higher complication rate for the ST group. More recent studies reveal a generally high patient satisfaction after simple elbow dislocation, regardless of the treatment [3, 12, 13, 27]. The authors agree, thus 95.5% of the patients achieved “excellent” outcome according to the MEPS. Since several studies promoted early functional therapy rather than immobilization in a cast, due to persisting motion deficit, unsatisfying subjective results and a delayed interval to return to work [5, 9, 14, 15, 20], the CT group of the present study received immediate functional therapy following ligamentous elbow dislocation. Kerschbaum et al. [12] also using early functional therapy, found a residual increased valgus stress angulation and posterolateral translation on ultrasound compared to the unaffected side after conservative treatment in a case series of 10 patients. Their clinical scores achieved comparable results to the CT group of the present study, such as the MEPS with 91 ± 9 points and DASH with 4 ± 4 points. Nevertheless, the sample size and a missing control group are limiting the authors’ conclusion that anatomical ligamentous healing does not exist after conservative treatment. In a comparative study evaluating conservative vs. surgical treatment after simple elbow dislocation in 54 patients, Krticka et al. [13] conclude that conservative treatment (*n* = 28) when applied at stable elbows (no re-instability within the arc of motion of 45°–120°) leads to statistically better results in terms of ROM and scores such as the MEPS, Oxford elbow score (OES) and the Quick-DASH. The MEPS reached 97 points (range:75–100) for conservative and 87.7 points (range: 60–100) for surgical treatment, whereas the Quick-DASH was 2.5 (range: 0–13.6) and 8.3 (0–27.3), respectively. Furthermore, the complication rate of the surgical group was statistically higher compared to the conservative group. In the present study, a higher complication rate without differences in the assessed clinical scores was observed. Moreover, two patients who were treated conservatively developed chronic instability and received ligament reconstruction and therefore had to be excluded. Consequently, the complication rate of the conservative group might be underestimated in the presented cohort. Krticka et al., reported short term results of 26 months and 32 months, yet, it is not known whether a longer follow-up period and objective measurements of joint congruence might have affected the results. A recently published study by Willin et al. [27] with a small sample size of 14 patients also investigated for ultrasound stability following either non-operative or surgical ligamentous repair in simple elbow dislocations. A significantly increased medial joint gapping was found for surgically treated patients. However, the study may have been underpowered, and the bias of laxity was no considered in the ultrasound measurements.

Surgical therapy has been promoted especially for active patients with high demands concerning sportive activities and work. In the present study we also see that 21 out 23 patients performed sports before elbow dislocation. Until now, there was a lack of studies, investigating return to sports rate and interval as well as frequency, satisfaction and pain level while performing sports following simple elbow dislocation. In the present study, ST patient returned to sports significantly faster compared to CT patients. Furthermore, a lower incidence of pain and higher capability and satisfaction performing sports was found. However, a relevant selection bias must be mentioned since patients with high functional demands were more likely to receive capsule-ligamentous repair and tendon refixation. It also should be considered that this special group of patients might be more motivated to return to sports and to work.

### Limitations

There are several limitations in this study. First, the retrospective study design omitted randomization. The assignment to CT and ST group was based on the stability of the elbow joint after initial reduction, severity of injury and the patient’s physical demands. This selection bias renders a definite conclusion on the preferable treatment option impossible. The severity of injury included in some cases the assessment of soft tissue damage seen in posttraumatic MRIs. However, only 9 out 21 patients received MRI scans in group CT whereas 23 out 23 received MRI scans in group ST. A relevant selection bias should be mentioned; thus, patients with a relevant soft tissue damage might be more likely to be treated operatively.

Second, due to the limited number of patients and respective data, influencing factors such as injury patterns, surgical refixation techniques and physical therapy could not be correlated with clinical or ultrasound results. Third, only varus- and valgus stress tests were performed for the ultrasound evaluation. Ultrasound measurements for posterolateral instability (PLRI) or posterolateral translation were not included. Nevertheless, residual PLRI can also lead to a subjective feeling of instability and therefore affecting the subjective results. Last but not least, the follow-up rate was quite low but the sample size is comparable to already published studies[10, 13]. Nevertheless, there are certain strengths of the present study including an a priori power calculation and a mid-term follow-up of 61.5 months, as well as the objective measurements of joint stability by ultrasound in a standardized fashion. A bias caused by individual laxity should be minimized due to comparison of measurements to the contralateral joint.

## Conclusion

Conservative and surgical treatment of patients with simple elbow dislocation leads to high patient satisfaction and good-to-excellent clinical results. Ultrasound evaluation revealed a stable elbow joint regardless of the chosen treatment. The subjective feeling of joint stability did not correlate with the ultrasound findings. Surgically treated patients returned faster to their previously performed sports. Thus, individual factors as well as the patient's demand should be considered when choosing the appropriate treatment. However, a higher complication rate may be expected for surgically managed patients.
